# Correlation between Saliva and Plasma Levels of Endothelin Isoforms ET-1, ET-2, and ET-3

**DOI:** 10.1155/2015/828759

**Published:** 2015-04-20

**Authors:** Roma Gurusankar, Prem Kumarathasan, Anusha Saravanamuthu, Errol M. Thomson, Renaud Vincent

**Affiliations:** ^1^Inhalation Toxicology Laboratory, Environmental Health Science and Research Bureau, Health Canada, Ottawa, ON, Canada K1A 0K9; ^2^Analytical Biochemistry and Proteomics Laboratory, Environmental Health Science and Research Bureau, Health Canada, Ottawa, ON, Canada K1A 0K9

## Abstract

Although saliva endothelins are emerging as valuable noninvasive cardiovascular biomarkers, reports on the relationship between isoforms in saliva and plasma remain scarce. We measured endothelins in concurrent saliva and plasma samples (*n* = 30 males; age 18–63) by HPLC-fluorescence. Results revealed statistically significant positive correlations among all isoforms between saliva and plasma: big endothelin-1 (BET-1, 0.55 ± 0.27 versus 3.35 ± 1.28 pmol/mL; *r* = 0.38, *p* = 0.041), endothelin-1 (ET-1, 0.52 ± 0.21 versus 3.45 ± 1.28 pmol/mL; *r* = 0.53, *p* = 0.003), endothelin-2 (ET-2, 0.21 ± 0.07 versus 1.63 ± 0.66 pmol/mL; *r* = 0.51, *p* = 0.004), and endothelin-3 (ET-3, 0.39 ± 0.19 versus 2.32 ± 1.44 pmol/mL; *r* = 0.75, *p* < 0.001). Correlations of BET-1, ET-1, and ET-3 within each compartment were positive in both plasma (*p* < 0.05) and saliva (*p* ≤ 0.1), whereas ET-2 was not significantly correlated with other isoforms in either plasma or saliva. For all isoforms, concentrations varied on average fivefold between individuals (90th/10th percentiles); individuals with high plasma endothelin levels generally had high saliva endothelin levels. Our results reveal that salivary ET isoform profiles portray the plasmatic profiles and support the view of coordinated regulation of ET-1 and ET-3, but distinct regulatory pathways for ET-2.

## 1. Introduction

As a diagnostic fluid, saliva has several advantages over blood [1, 2]. Saliva is inexpensive and easier to collect, and sufficient volume can be obtained to allow performance of a variety of analyses. For patients, particularly children, the noninvasive sample collection reduces anxiety and discomfort and simplifies procurement of repeated samples for time-series analyses. Saliva contains a wide array of proteins and peptides that are responsive to pathological conditions [3]. Advanced instrumentation and refined analytical techniques have been successfully applied for discovering oncological [4], hormonal [5], immunological [6], and cardiovascular [7, 8] biomarkers that can be informative for early detection and assessment of progression of oral and systemic diseases.

Of particular interest, saliva is known to contain detectable levels of endothelins, an important risk marker for cardiovascular disease [9]. Endothelins are a family of potent vasoconstrictor peptides consisting of three distinct isoforms, endothelin-1 (ET-1), endothelin-2 (ET-2), and endothelin-3 (ET-3) coded by distinct genes [10]. The mature endothelins are produced through cleavage of the big endothelin (BET) precursors by endothelin-converting enzymes [11, 12]. Endothelin-1, the most studied isoform, has been implicated in several diseases, particularly in the progression of cardiovascular diseases [13, 14]. It has been known for two decades that ET-1, ET-2, and ET-3 are present in saliva [15, 16]. However, only recently have levels of saliva ET-1 been related to conditions such as chronic heart failure [17], upper gastrointestinal diseases [18], vibration-induced white finger [19], and oral cancer [20, 21]. The relationship between saliva and plasma ET-2 and ET-3 isoforms is comparatively less well understood. Because of the emerging significance of all three isoforms in health and disease, notably the role of ET-2 in the cardiovascular system, in ovulation, immunology, and cancer [22], we sought to extend the data on the relationships between the three endothelin isoforms in concurrent saliva and plasma samples.

## 2. Materials and Methods

### 2.1. Reagents

Ethylenediaminetetraacetic acid (EDTA), trifluoroacetic acid (TFA), phenylmethyl sulfonyl fluoride (PMSF), 3,4-dichloroisocoumarin, molecular weight cut-off filters (30 kDa), endothelin-1 (ET-1, human), endothelin-2 (ET-2, human), and endothelin-3 (ET-3, human) were obtained from Sigma Aldrich (Oakville, Ontario). Big endothelin-1 (BET-1, human) was obtained from Bachem Bioscience (American Inc., CA, USA). Acetonitrile, acetone, methanol, and hydrochloric acid were purchased from Sigma Aldrich (Oakville, Ontario). Amber glass vials and screwcaps with septa were purchased from Chromatographic Specialities (Brockville, Ontario). Deionized water was obtained from a Super-Q Plus high purity water system (Millipore Corporation, Bedford, MA). Compressed gaseous nitrogen was of UHP grade quality and was supplied by Matheson Gas Products (Whitby, Ontario).

### 2.2. Biological Samples

Anonymous, paired human plasma and saliva samples (from males) certified free of HIV, HEP-A, and HEP-B were purchased commercially (Innovative Research Inc., MI). Saliva samples were collected by spitting in a sterile cup, without stimulation. Both plasma and saliva samples were treated on site with PMSF (final, 1.7 mg/mL) and EDTA (final, 10 mg/mL) to stabilize endothelins [23], shipped to our laboratory in dry ice, and stored at −80°C until further use. Saliva samples containing phlegm or low volume of fluid were discarded. Thirty (30) sample pairs of good quality and in sufficient amounts for analysis were retained for this study.

### 2.3. Extraction of Endothelins from Plasma and Saliva

Plasma and saliva endothelins were extracted following Kumarathasan et al. [23]. Briefly, plasma (250 *μ*L) and saliva (1 mL) samples were treated with ice-cold 3,4-dichloroisocumarin solution in isopropanol to prevent conversion of BET-1 to ET-1 during the sample processing. The samples were deproteinized with ice-cold acid-acetone mix (acetone : 1 N HCl : water, 40 : 1 : 5) and centrifuged at 9000 ×g for 10 min, and the supernatants obtained were concentrated by evaporation under nitrogen flow. Deproteinization and concentration stages were repeated once more to ensure the removal of abundant proteins. Samples were then loaded onto 30 kDa molecular weight cut-off filters (prewashed with deionised water) and centrifuged at 5000 ×g for 30 min. These filters were then washed with 50% methanol and centrifuged at 5000 ×g for 30 min. Filtrates were dried under nitrogen and reconstituted in 75 *μ*L of 30% acetonitrile in 0.2% TFA/H_2_O for HPLC-fluorescence analysis.

### 2.4. HPLC-Fluorescence Analysis of Endothelins

The HPLC unit consisted of a Gilson solvent delivery system (Mandel Scientific, Guelph, ON), a Gilson autosampler (model 231 XL; Middleton, WI), a Supelcosil LC-318 reverse-phase column (25 cm length, 4.6 mm id, 5 *μ*m particle size, and 300 Å pore dimension; Supelco, Oakville, ON), and a RF 551 model fluorescence detector (Shimadzu, Japan). Endothelins (20 *μ*L injection volume) were separated using a gradient elution with acetonitrile/water mobile phase at a flow rate of 1 mL/min and detected at ***λ***_EX_ 280 nm and ***λ***_EM_ 340 nm [23]. Blanks were analyzed after each set of four samples in order to assess the extent of analyte carryover.

### 2.5. Statistical Analyses

Student's *t*-test or Mann-Whitney Rank Sum Test was carried out as appropriate using SigmaStat v 11.0 (SPSS Inc., Chicago, IL). Results are presented as mean ± standard deviation. Correlation between different endothelin isoforms in plasma and saliva was tested using Pearson's Product Moment correlation revealing *p* and *r* values. Statistical significance was accepted for *α* = 0.05.

## 3. Results and Discussion

**Figure 1:**
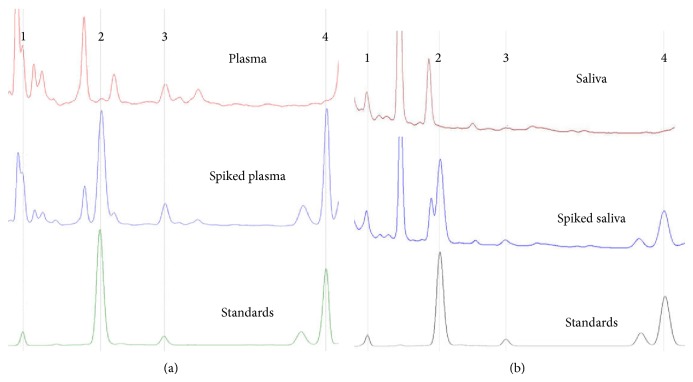
HPLC profiles of endothelin isoforms ET-3 (1), BET-1 (2), ET-1 (3), and ET-2 (4) in human (a) plasma and (b) saliva samples (including unspiked and spiked biological matrices along with endothelin standards).

We used HPLC-fluorescence to measure simultaneously the isoforms BET-1, ET-1, ET-2, and ET-3 (Figures 1(a)-1(b)) in time-matched plasma and saliva sample pairs (*N* = 30) obtained from anonymous individuals (Table 1). Our results confirm and extend previous reports of the presence of endothelins in saliva [15, 16] and of a relationship between saliva and circulating endothelins [17–19], and the correlated measurements provide additional insight into the relationship of ET-2 to the other two isoforms [22]. The recoveries, analytical precision, and accuracy of the HPLC-fluorescence procedure [23] are comparable to values reported by Walczak et al. for HPLC with electrospray tandem mass spectrometry detection [24]: recoveries of endothelins from spiked plasma are between 60% (ET-2) and 97% (ET-1), depending on the endothelin isoforms; analytical precision is on the order of ±4% for replicate peptide standards; analytical accuracy is ±20% for replicate measurements of plasma samples; limit of detection is 0.2–0.5 pmol; and linearity is 1–100 pmol on the column (20 *μ*L injection volume). Both methods detect the separated peptides directly (i.e., on the basis of autofluorescence of aromatic amino acids by HPLC [23, 25] or from mass ion fingerprints in MS/MS [24]), which is different from ELISA detection of immunoreactive endothelin in total plasma [26, 27]. For this reason, we have verified identities of analytes by pulling down of ET-1 and ET-3 with monoclonal antibodies during sample processing, confirming the correspondence between immunoreactive endothelins in plasma and the peptides measured by direct autofluorescence detection after separation on column [23].

It should be noted that the aim of our study was not to explore the physiological significance of variations of saliva and plasma endothelin levels, within and between individuals, and for this reason we have not requested any information on the health status, cardiovascular or buccal, of the donors. Salivary secretions and saliva volume will be affected by water and food uptake and by medication. These factors, together with the health status of subjects and sample quality, can contribute to the variance in observed saliva endothelin concentrations and relationship to plasma levels. Furthermore, sample collection procedures, sample preparation methods, matrix effects, and the analytical approach used are likely to impact the absolute values of estimates. Finally, because all samples analyzed for endothelin content in our small study were from male donors, our data do not address potential gender differences.

**Figure 2:**
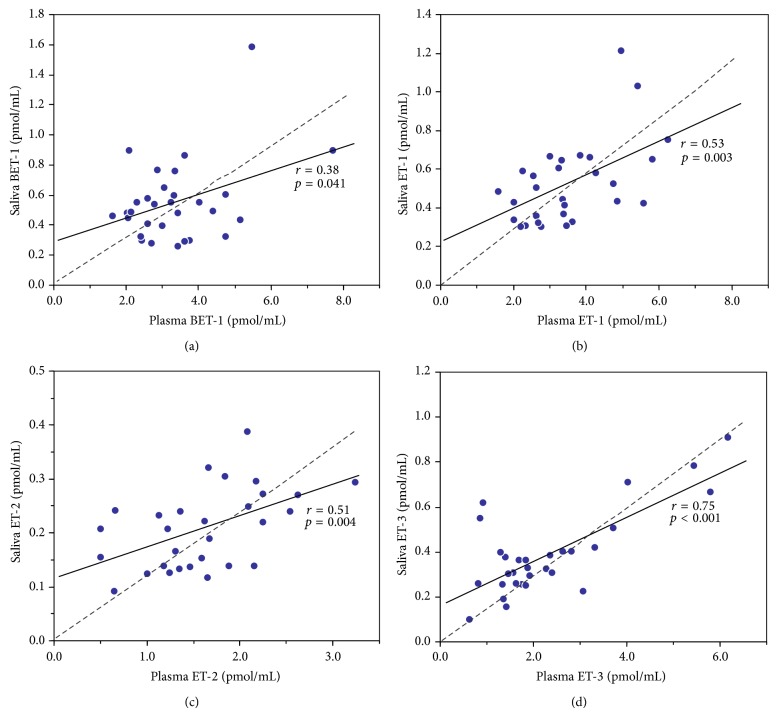
Correlation between endothelin isoforms BET-1 (a), ET-1 (b), ET-2 (c), and ET-3 (d) in plasma and saliva of male subjects (*n* = 30) measured by HPLC-fluorescence. Assuming simple linearity, the linear regression (solid line) intercept on saliva axis is an estimate of salivary gland endothelin production (*r* and *p* values indicated). The linear regression forced through zero (dashed line) assumes diffusion of endothelins entirely from plasma.

Notwithstanding those limitations, our observations reveal that levels of all endothelin isoforms were significantly correlated between the saliva and plasma matrices in the subjects studied: BET-1 (*r* = 0.38, *p* = 0.041), ET-1 (*r* = 0.53, *p* = 0.003), ET-2 (*r* = 0.51, *p* = 0.004), and ET-3 (*r* = 0.75, *p* < 0.001) (Table 1, Figure 2). Our results indicate a slight increase in the ratios of endothelin levels in plasma versus saliva with age of the subjects (ET-1, plasma/saliva ratio in 18–40 years versus 41–63 years, 6.11 ± 2.16 versus 8.67 ± 2.51, *p* = 0.006), which is potentially attributable to saliva osmolality changes with age [28, 29]. Interestingly, the relationship between osmolality changes and endothelin system has been previously reported [30]. Endothelin levels varied on average by fivefold between individuals, and this applied to all four isoforms measured, in plasma as well as saliva (90th/10th percentiles). In general, high BET-1 and ET-1 levels were predictive of high ET-3 levels within an individual (Table 2). Within plasma, a significant positive correlation was seen for BET-1 versus ET-1 (*r* = 0.39, *p* = 0.033), BET-1 versus ET-3 (*r* = 0.46, *p* = 0.011), and ET-1 versus ET-3 (*r* = 0.57, *p* = 0.001). Within saliva, a significant positive correlation was seen for BET-1 versus ET-1 (*r* = 0.75, *p* < 0.001). Correlations between BET-1 versus ET-3 (*r* = 0.31, *p* = 0.091) and ET-1 versus ET-3 (*r* = 0.27, *p* = 0.144) in saliva were not significant. There were no significant correlations between ET-2 and other isoforms in either plasma or saliva.

The origin of saliva endothelins is not well established. Whole saliva contains secretions from the major parotid, submandibular and sublingual glands, the palate, buccal and labial mucosa, and so on [31]. Expression of preproET-1 and preproET-3 as well as the ET_A_ and ET_B_ receptors has been detected by RT-PCR analyses of submandibular glands of rats [32]. Endothelin-1 has also been detected in striated duct cells of human salivary glands by immunohistochemical analysis [33]. Therefore, it is plausible that endothelins are secretory products from salivary glands. However, serum molecules that are not part of the normal salivary secretory constituents, such as proteins, drugs, and hormones, can also reach saliva by passive diffusion [1, 2, 34–36]. We have measured six- to eightfold higher concentrations of endothelins in plasma by comparison to saliva, and hence the concentration gradient may result in diffusion of the peptides from the capillaries of the mucosa into the salivary fluid. If the intercept of the linear regressions of saliva versus plasma endothelin concentrations is taken as an estimate of the contribution by salivary glands, then at least half (BET-1, ET-1, and ET-2) to two-thirds (ET-3) of the salivary endothelins may originate from plasma (Figure 2).

Correlation among the different endothelin isoforms within individuals and the large variance of endothelin levels between individuals may be due to a number of factors, including common regulatory mechanisms, genetic polymorphisms, and physiological status. Correlation between BET-1 and ET-1 should be expected because of the direct precursor-product relationship [37]. Furthermore, endothelin converting enzyme-1, which cleaves BET-1 to ET-1, shares some regulatory elements with the preproET-1 gene, such that when preproET-1 is transcriptionally activated, ECE-1 tends to be activated as well [38]. In healthy individuals, ET-1 plays an important role in the modulation of vasomotor tone, in conjunction with nitric oxide, but also in regulating cellular proliferation and differentiation in tissues during growth, development, and repair [39, 40].

**Figure 3:**
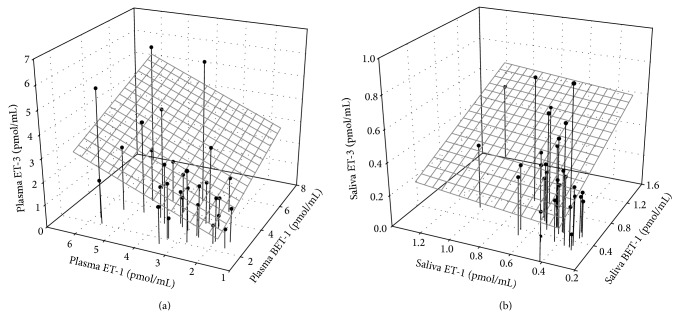
Correlation between BET-1, ET-1, and ET-3 isoforms in plasma (a) and saliva (b) of male subjects.

There is good evidence for coordinated regulation of ET-1 and ET-3. In individuals with high basal ET-1 production, activation of preproET-3 expression through a feedback control mechanism may compensate for the pressor effects of the high circulating levels of ET-1. Endothelin-3 stimulates nitric oxide synthase expression and NO production through binding to the ET_B_ receptor [41], and increase of ET-1 with a decrease of ET-3, due to endothelial dysfunction, has been reported in patients with pulmonary arterial hypertension of various etiologies [42, 43]. It is also possible that high or low transcriptional activity of both preproET-1 and preproET-3 genes within an individual is determined by genetic polymorphism or epigenetic conditioning of transacting elements with common cis-acting regulatory sequences between the two genes, but this remains to be investigated. In our analyses, the strongest associations were seen between saliva ET-3 and plasma ET-3 (*r* = 0.75, *p* < 0.001) and between saliva BET-1 and saliva ET-1 (*r* = 0.75, *p* < 0.001). An overall relationship between ET-3 and BET-1 and its mature product ET-1 was observed in plasma and saliva but was stronger in plasma (Figure 3). The similar ratio of BET-1 to ET-1 in plasma (3.35 versus 3.45 pmol/mL) and saliva (0.55 versus 0.52 pmol/mL) suggests a relative stability of the peptides during sampling and sample processing. However, although saliva ET-2 correlated with plasma ET-2 (*r* = 0.51, *p* = 0.004), the poor correlation between ET-2 and the other endothelin isoforms, within plasma (positive, *p* > 0.05) and within saliva (negative, *p* > 0.05), is in line with the concept of ET-2 regulatory pathways distinct from those of ET-1 and ET-3 [22].

In conclusion, our data confirm an overall positive correlation between plasmatic and salivary BET-1, ET-1, ET-2, and ET-3 levels and between the BET-1, ET-1, and ET-3 within each of the two compartments. The isoform ET-2 correlates poorly with ET-1 and ET-3, within saliva and within plasma. Salivary ET isoform profiles portray the plasmatic profiles and support the view of coordinated regulation of ET-1 and ET-3, but distinct regulatory pathways for ET-2.

## Figures and Tables

**Table 1:** Plasma and saliva endothelin levels (mean ± SD) in male subjects.

	BET-1	ET-1	ET-2	ET-3	Total	BET-1/ET-3
Plasma (pmol/mL)						
All (30)	3.35 ± 1.28	3.45 ± 1.28	1.63 ± 0.66	2.32 ± 1.44	10.8 ± 3.46	1.84 ± 1.00
18–40 years (19)	3.39 ± 1.38	3.22 ± 1.43	1.56 ± 0.61	1.97 ± 1.21	10.1 ± 3.7	2.10 ± 1.07
41–63 years (11)	3.29 ± 1.15	3.87 ± 0.89	1.77 ± 0.73	2.91 ± 1.67	11.8 ± 2.9	1.38 ± 0.70
				*P* = 0.039		*P* = 0.043

Saliva (pmol/mL)						
All (30)	0.55 ± 0.27	0.52 ± 0.21	0.21 ± 0.07	0.39 ± 0.19	1.68 ± 0.53	1.61 ± 0.75
18–40 years (19)	0.59 ± 0.32	0.56 ± 0.25	0.20 ± 0.07	0.37 ± 0.16	1.71 ± 0.64	1.68 ± 0.66
41–63 years (11)	0.49 ± 0.12	0.47 ± 0.13	0.23 ± 0.09	0.43 ± 0.23	1.62 ± 0.27	1.48 ± 0.91

Plasma/saliva						
All (30)	6.94 ± 3.30	7.05 ± 2.58	8.18 ± 3.16	6.15 ± 2.34	6.67 ± 1.79	
18–40 (19)	6.86 ± 3.50	6.11 ± 2.16	8.16 ± 3.18	5.71 ± 2.73	6.30 ± 1.93	
41–63 (11)	7.07 ± 3.10	8.67 ± 2.51	8.22 ± 3.28	6.89 ± 1.25	7.31 ± 1.36	
		*P* = 0.006				

Note: *P* value for comparison between the two age groups (*t*-test or Mann-Whitney Rank Sum Test). The number of subjects is shown in parentheses.

**Table 2:** Pearson product moment correlation (*r*-value) for endothelin isoforms in plasma (PL) and saliva (SL) of male subjects (*n* = 30).

	ET-1 PL	ET-2 PL	ET-3 PL	BET-1 SL	ET-1 SL	ET-2 SL	ET-3 SL
BET-1 PL	0.39 (0.033)	0.27 (0.157)	0.46 (0.011)	0.38 (0.041)	0.30 (0.110)	−0.11 (0.571)	0.41 (0.024)
ET-1 PL		0.25 (0.183)	0.57 (0.001)	0.31 (0.093)	0.53 (0.003)	−0.22 (0.251)	0.46 (0.011)
ET-2 PL			0.13 (0.511)	0.01 (0.974)	0.15 (0.434)	0.51 (0.004)	0.16 (0.406)
ET-3 PL				0.17 (0.383)	0.16 (0.409)	−0.29 (0.121)	0.75 (<0.001)
BET-1 SL					0.75 (<0.001)	−0.12 (0.534)	0.31 (0.091)
ET-1 SL						−0.13 (0.491)	0.27 (0.144)
ET-2 SL							−0.167 (0.378)

Note: *P* values are given in parentheses.

## Abbreviations

ET: Endothelin
ECE: Endothelin-converting enzyme
EDTA: Ethylenediaminetetraacetic acid
HEP-A: Hepatitis A
HEP-B: Hepatitis B
HIV: Human immunodeficiency virus
PMSF: Phenylmethylsulfonyl fluoride
TFA: Trifluoroacetic acid.
